# Beyond the Bench: Cultivating Environmental Leadership in the Midwest

**DOI:** 10.1289/ehp.113-a596

**Published:** 2005-09

**Authors:** Tanya Tillett

Today’s youth are the environmental health leaders of tomorrow. The Environmental Health Sciences Research Center (EHSRC) at The University of Iowa, in conjunction with its partner, the Belin–Blank International Center for Gifted Education and Talent Development (also a component of The University of Iowa), is helping some of these future leaders understand the environment and their role in it, with the goal of inspiring the next generation of environmental health advocates.

Each summer since 1997, the two partners have joined forces to conduct the Environmental Health Sciences Institute for Rural Youth (EHSI), an intensive, full-scholarship, one-week residential experience for rising tenth-graders from small, rural Midwest communities. By giving high school students access to a wealth of environmental health information and helping them translate that information for dissemination to their own communities, the EHSI helps foster leadership qualities that will help them apply those skills to their future careers and their personal lives.

Each summer the EHSI accepts about 15 students to the program, and houses them in student residence halls on the Iowa campus. According to David Osterberg, director of the EHSI, the primary goal of the program is to inspire students to consider the environmental health sciences as a possible future career.

“We have students for a week, so we can aspire to do many things,” he says. “We help develop mentoring relationships between smart high school students and our scientists, expose students to cutting-edge research, show them a full range of environmental health topics, and give them some career options. I especially like to emphasize policy so students realize there are potential solutions to problems that impact the environment and human health.”

Throughout the week, the students are exposed to information on environmental health and related research through lectures, interactive lab sessions, one-on-one mentoring, and field trips. In this year’s session, students attended lectures on such diverse topics as the relationship between cancer and the environment, nanotechnology in environmental health science, global climate change, and the connection between agriculture and health. The mentoring and lab sessions then give the students a first-hand glimpse of current research related to the lecture content.

This summer’s lab activities included a pathology session in which students examined specimens of human organs to compare cancerous and healthy tissues. Another session focused on inhalation toxicology. Students dissected mouse lung tissue and examined the cells under a microscope to determine the effects of grain dust exposure on the lung. Afterwards, they watched a dust measurement and quantification demonstration in the EHSRC’s environmental modeling and assessment facilities.

The program also exposes the students to initiatives taking place in the Iowa community that encourage environmental responsibility. One of this year’s field trips was a visit to the Amana Lily Pond, a wetland that has been planted with poplar trees to act as natural filters to prevent herbicides, insecticides, and fertilizers from entering the creek and emptying into the pond. The students also saw a demonstration by the Iowa Renewable Energy Association of the “solar traveler,” a mobile demonstration unit that produces electricity via solar power.

Since public speaking is a crucial skill for scientists and public health workers, the program also includes a session on speaking in front of groups that helps the students improve their body language and voice projection to deliver an effective presentation. Once the summer session ends, each student chooses an environmental heatlh science topic and organizes information learned over the week into a presentation that is delivered to a school group and to a community group in their hometown.

The presentations, as well as the other aspects of the EHSI experience, give the students the opportunity to become environmental health science ambassadors who can potentially impact the lives of their families and neighbors. “We hope that in the course of EHSI Week, students gain an appreciation for environmental issues as well as their own personal stake in how these issues affect their health, their families, and their communities,” says Osterberg.

## Figures and Tables

**Figure:**
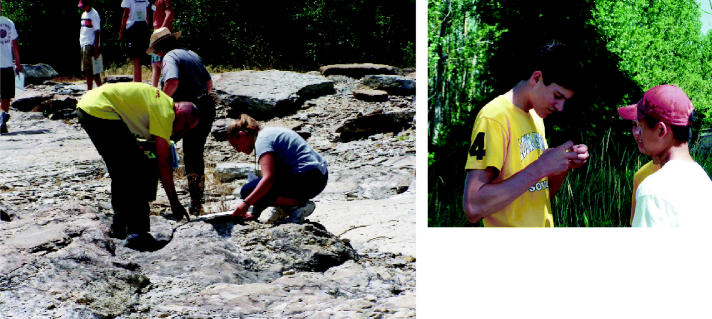
Budding environmental scientists. (left) EHSI students explore the Devonian Fossil Gorge in Coralville, Iowa. A 1993 flood washed away layers of earth and exposed bedrock containing a multitude of fossils. The field trip followed a lecture on effects of global climate change. (above) Students perform a chemical analysis on water collected at the Amana Lily Pond phytoremediation site.

